# Tracheobronchial endoluminal hamartochondroma resected via rigid bronchoscopy: 2 case reports of a rare entity

**DOI:** 10.12688/f1000research.159445.1

**Published:** 2025-01-14

**Authors:** Amany Touil, Maazaoui Sarra, Darragi Karim, Chaabane Mariem, Znegui Tasnim, Ayadi Rahma, Fitouhi Nizar, Baccouche Ines, Racil Hager, Chaouch Nawel

**Affiliations:** 1 Department of Respiratory Diseases and Interventional Endoscopy, Pavillon 2, Abderrahmen Mami Pneumology and Phthisiology Hospital, Ariana, Tunisia; 2 Faculty of Medicine of Tunis, Research Laboratory LR12SP04, Tunis El Manar University, Tunis, Tunis, Tunisia; 3 Department of Pathology, Abderrahmen Mami Pneumology and Phthisiology Hospital, Ariana, Ariana, Tunisia; 4 Department of Anesthesiology and Critical Care, Abderrahmen Mami Pneumology and Phthisiology Hospital, Ariana, Ariana, Tunisia; 5 Department of Radiology, Abderrahmen Mami Pneumology and Phthisiology Hospital, Ariana, Ariana, Tunisia

**Keywords:** Benign tumor, Tracheobronchial endoluminal hamartochondroma, Chondroid hamartoma, Rigid bronchoscopy, Flexible bronchoscopy, Diode laser

## Abstract

Tracheobronchial endoluminal hamartochondroma (HC) is a rare benign tumor, most frequently diagnosed in individuals between the sixth and seventh decades of life. The clinical presentation is usually very noisy. Here, we report two interesting cases. The first patient is a 58-year-old man who was wrongly treated for chronic obstructive pulmonary disease. HC was in the lower third of the trachea. The second patient is a 43-year-old man with a history of bronchiectasis and right lower lobectomy. HC was in the middle lobe bronchus. The resection via rigid bronchoscopy with diode laser was successful without any complications. Only one patient keeps a small stable tumor residue. The rigid bronchoscopy with laser-application in tracheobronchial endoluminal HC is safe and effective. Flexible bronchoscopy is important during the follow-up.

## 1. Introduction

Pulmonary hamartochondroma (HC) is the most common benign lung tumor; however, its tracheobronchial location is very rare. Endoscopic treatment is currently recommended as the first-line treatment for benign tracheobronchial tumors with several advantages, but it must be rapid to avoid irreversible parenchymal lesions. We report two cases of HC that were successfully treated with laser-assisted rigid bronchoscopy, a safe and effective therapeutic method.

## 2. Case presentation

### 2.1 Case report 1

**Figure 1.**
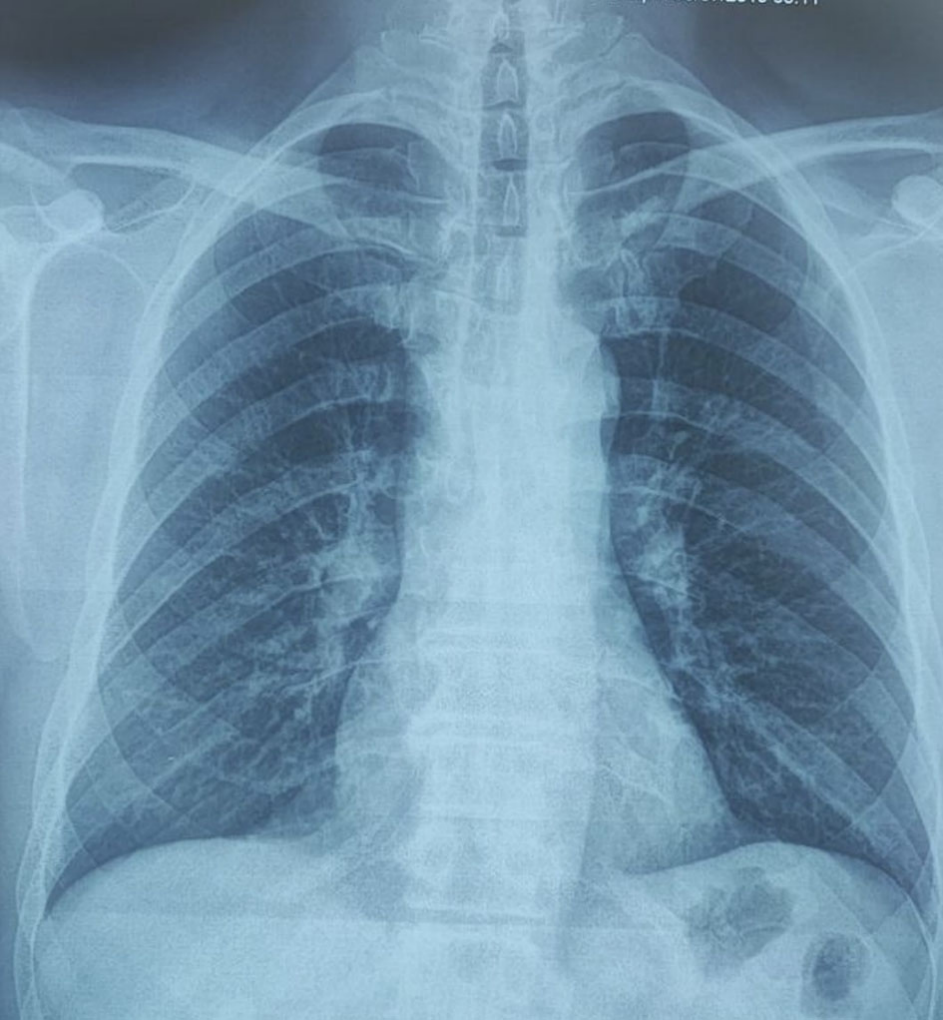
Chest radiography at admission: There was no significant abnormality.

**Figure 2.**
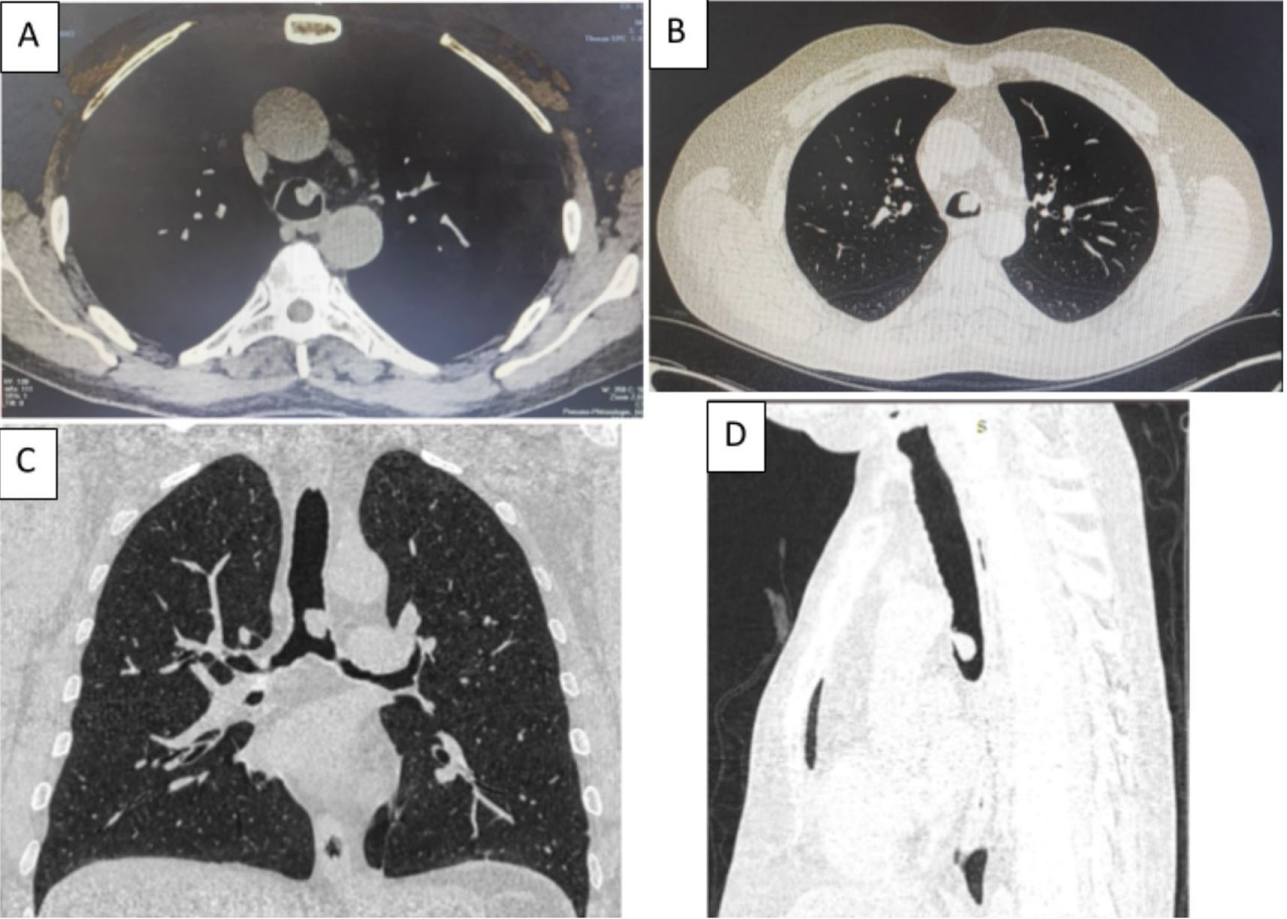
Chest CT-scan: Hypodense pedunculated budding lesion of the left antero-lateral tracheal wall located above the carina.

**Figure 3.**
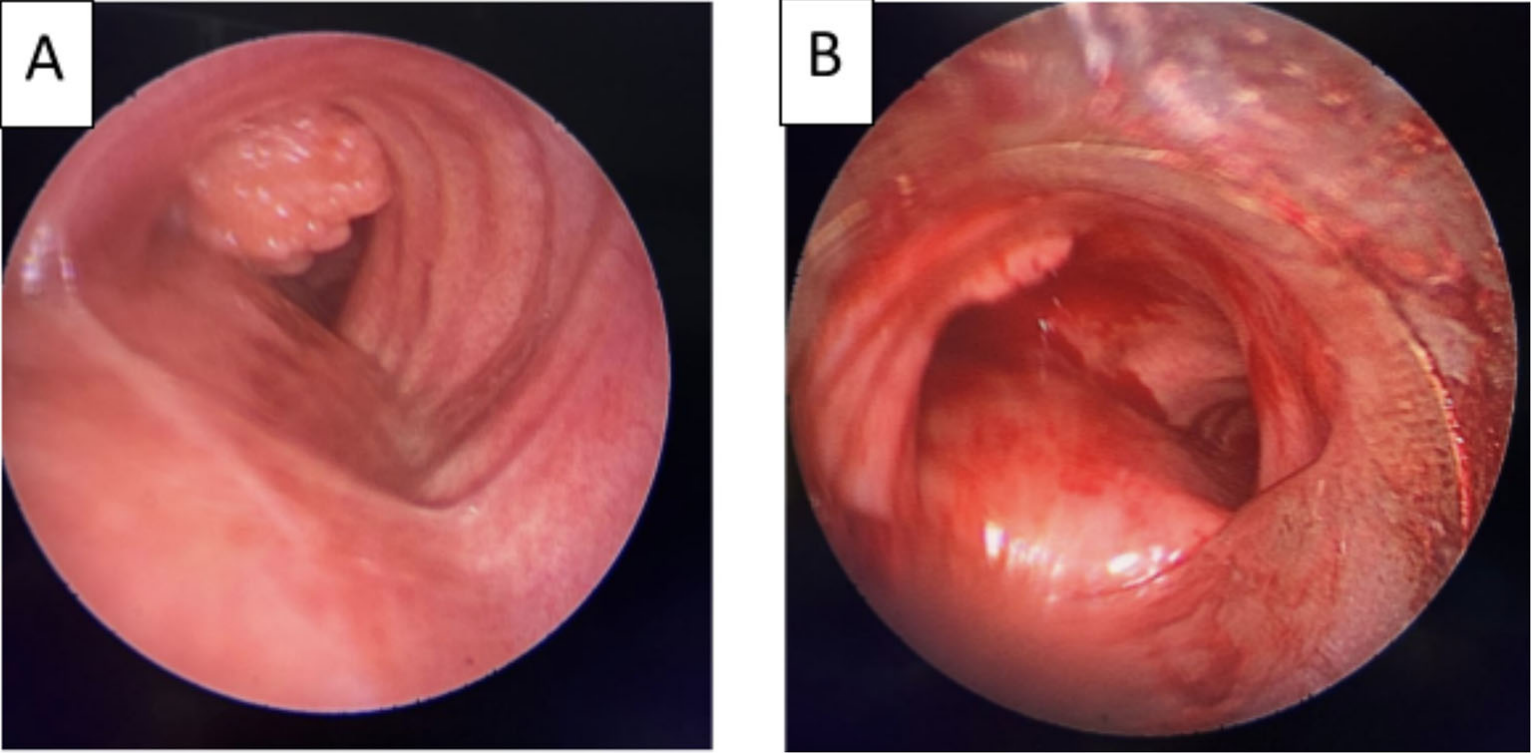
Rigid bronchoscopy findings. (A): A pale pink tumor at the lower third of the trachea. (B): A small tumor residue.

**Figure 4.**
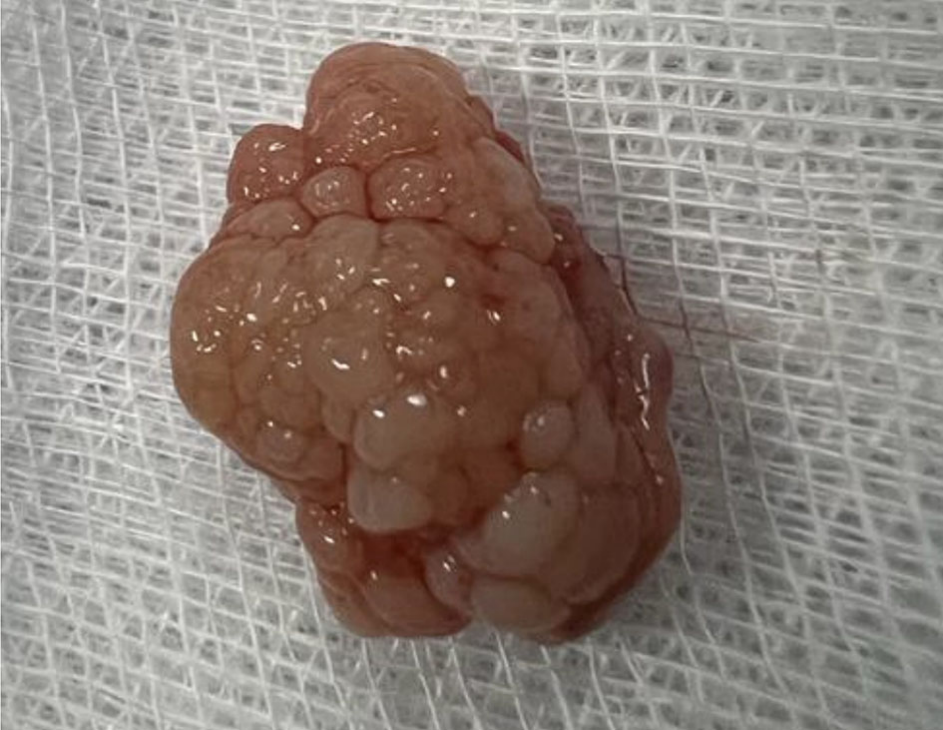
Macroscopic aspect: a non-vascularized pale pink budding tumor.

**Figure 5.**
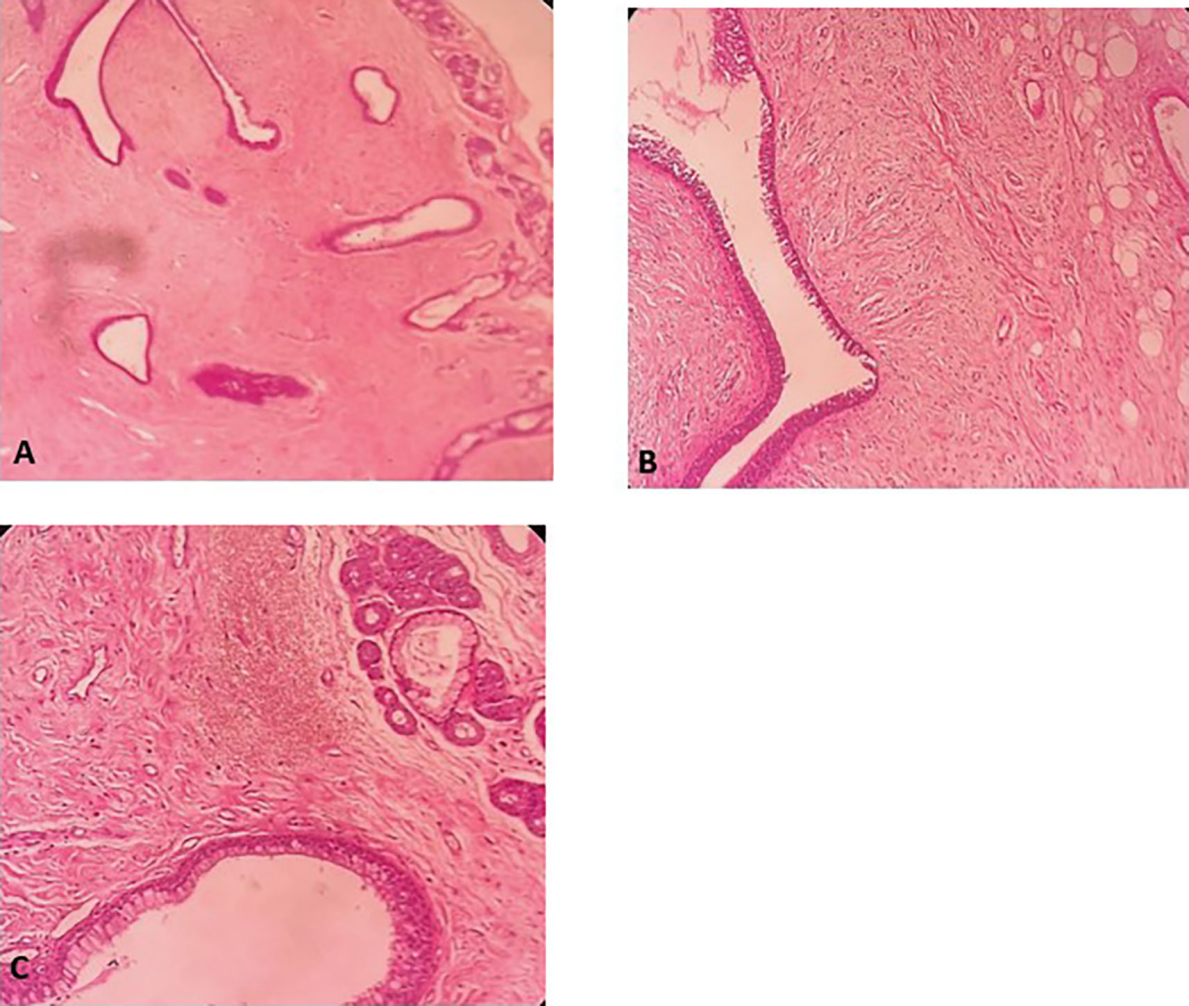
Histopathological examination. A: Hypertrophic seromucous gland admixed with variable amounts of fibro adipose tissue, some spindle cells and myxoid stroma. B: Fibrous adipose tissue, spindle cells, and myxoid stroma. C: Hypertrophic seromucous gland admixed with variable amounts of fibrous tissue, spindle cells and myxoid stroma.

**Figure 6.**
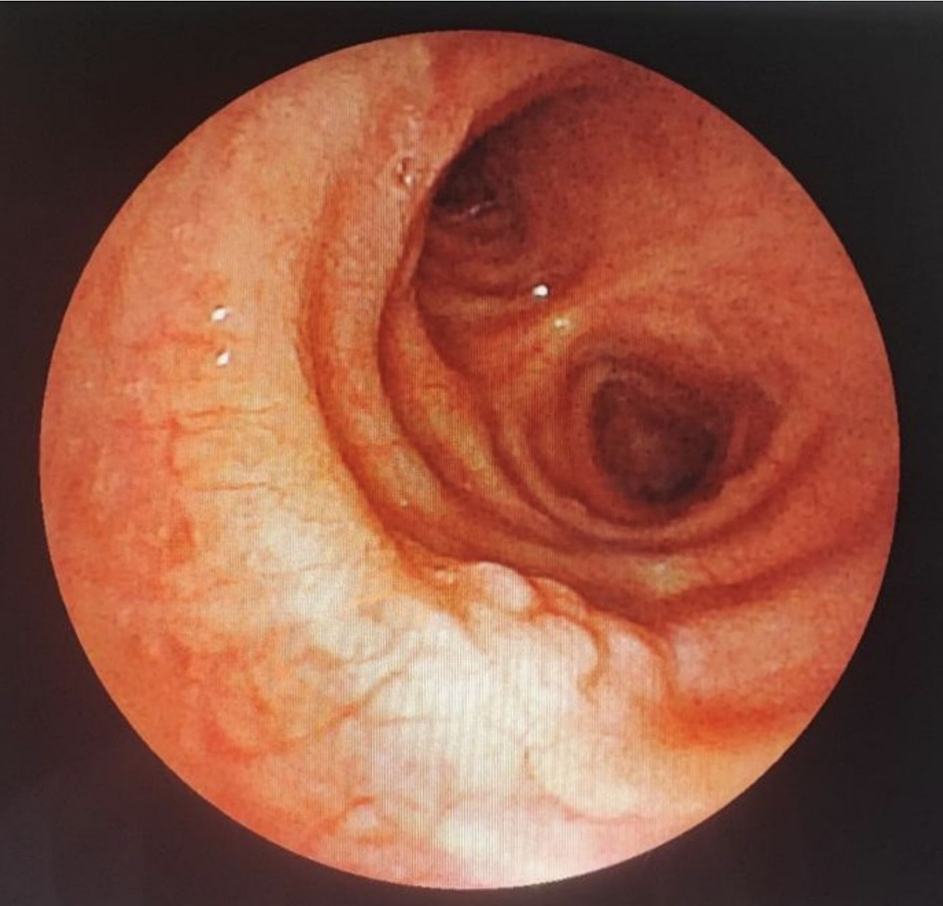
Flexible bronchoscopy findings: A stable tumor residue, not obstructing the tracheal lumen.

A 58-year-old male with a continued 70-pack-year smoking history was referred to our department for the endoscopic treatment of an endotracheal tumor. Four years previously, the patient presented with isolated shortness of breath and was treated for chronic obstructive pulmonary disease (COPD). Physical examination showed wheezes, and spirometry had met the GOLD criteria for the diagnosis of COPD with very severe airflow limitation (post-bronchodilator FEV1: 1.02 L (29%), post-bronchodilator FEV1/FVC ratio: 45%). The patient was subsequently hospitalized for bilateral hypoxemic pneumonitis and acute respiratory failure, which was treated as COPD exacerbation with a good evolution under usual treatment. On admission in our department, his clinical examination was normal, and his oxygen saturation was 97% (room air). Laboratory tests and chest radiography revealed no abnormalities ( Figure 1). Chest computed tomography (CT) scan showed a hypodense pedunculated budding lesion of the left anterolateral tracheal wall located just above the carina, 12 cm from the vocal cords and measuring 17 × 15 × 10 mm ( Figure 2). Flexible bronchoscopy confirmed an endotracheal lesion, but biopsies were non-contributory. Based on these findings, laser-assisted mechanical resection of the tumor has been validated as a feasible treatment option. Rigid bronchoscopy, performed under general anesthesia, revealed a non-vascularized pale pink tumor located at the lower third of the trachea approximately 1 cm distal to the carina, which obstructed 60% of the tracheal lumen ( Figure 3A). Following the initial exploration, the tumor was removed at the tip of the bronchoscope following laser treatment. On the final examination, a small tumor residue was observed ( Figure 3B). There were no complications, and the patient was rapidly discharged. The tumor measured 18 × 10 mm ( Figure 4). Histopathological examination of the samples stained with Hematoxylin and Eosin (H&E) showed the presence of hypertrophic seromucous gland admixed with variable amounts of fibrous adipose tissue, spindle cells, and myxoid stroma ( Figure 5). The diagnosis of endobronchial HC was established. The patient subsequently underwent follow-up flexible bronchoscopies, which all revealed that the tumor residue was stable and did not obstruct the tracheal lumen with a follow-up of three years ( Figure 6). The patient is currently asymptomatic, and his last spirometry examination revealed no abnormalities.

### 2.2 Case report 2

**Figure 7.**
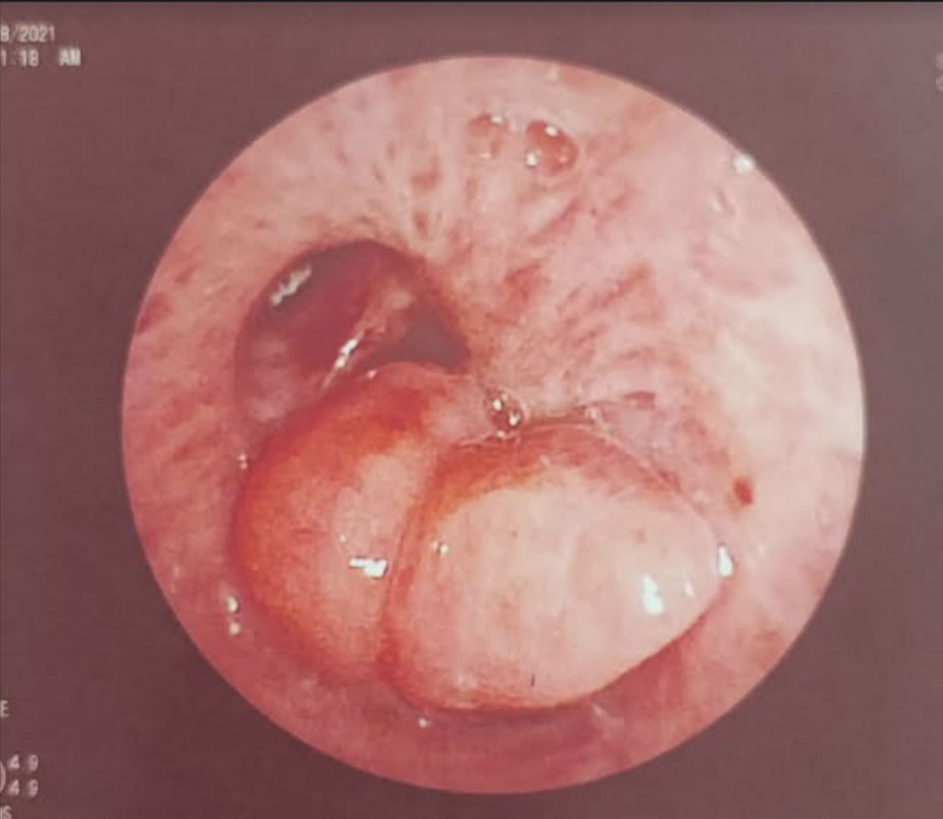
Flexible bronchoscopy findings: A yellowish polylobed budding formation obstructing completely the middle lobe bronchus.

**Figure 8.**
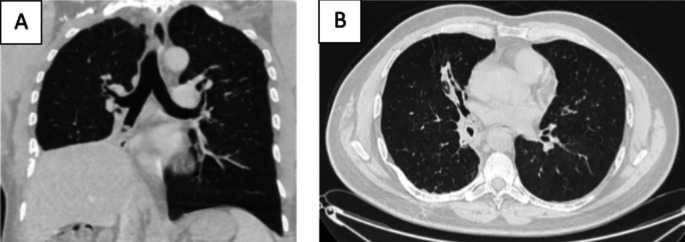
Chest CT-scan: Endobronchial lesion of fatty density in the middle lobe bronchus, responsible for minimal bronchiectasis.

**Figure 9.**
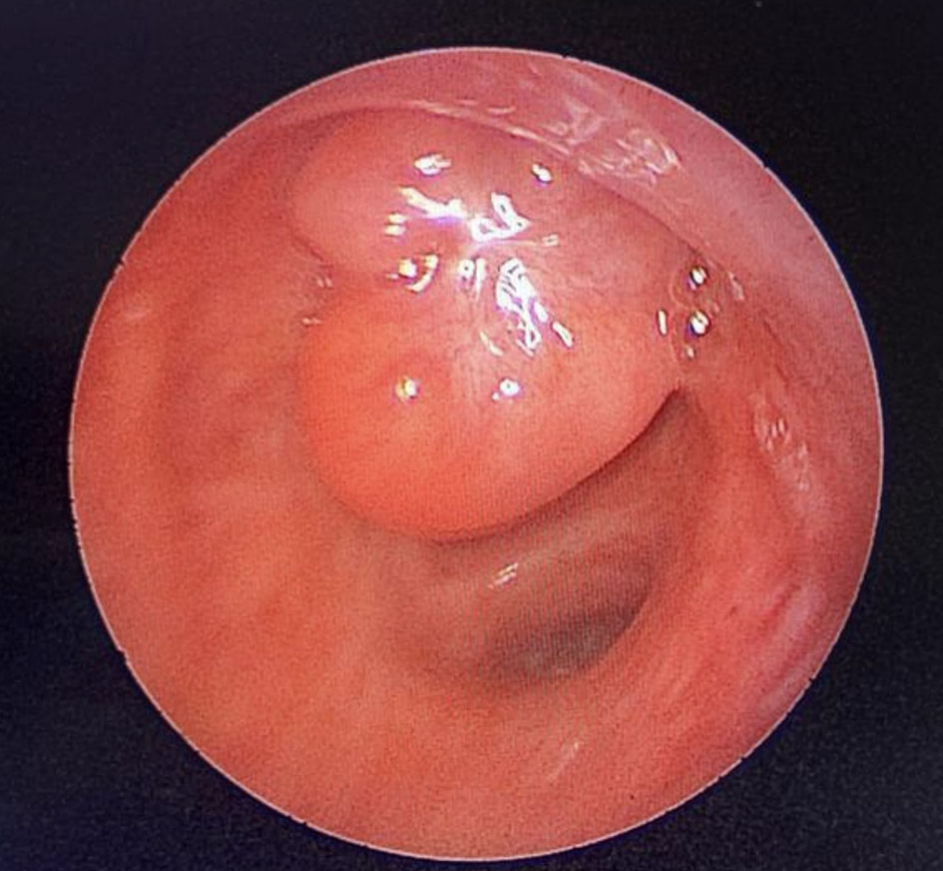
Rigid bronchoscopy findings: The tumor occludes the middle lobe bronchus and protrudes into the bronchus intermedius.

**Figure 10.**
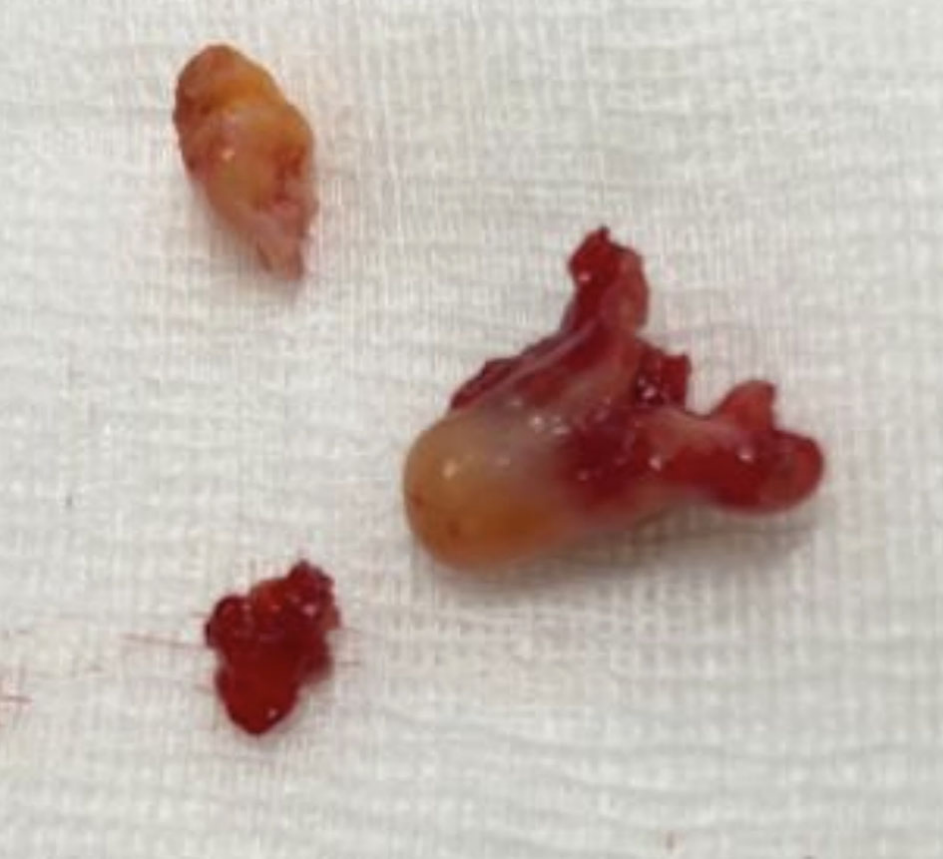
Macroscopic examination: 2 yellowish smooth fragments measuring 1.5*1.5*0.5 cm long axis.

**Figure 11.**
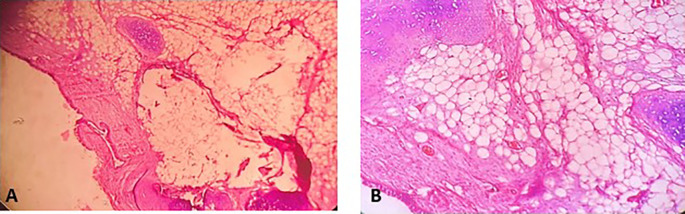
Histopathological examination. A: Hyaline cartilage admixed with variable amounts of fibro adipose tissue, some spindle cells and myxoid stroma. B: Nodules of hyaline cartilage interspersed with spindle cells and myxoid stroma.

A 43-year-old nonsmoker man was referred to our department for respiratory preparation before surgical excision of an endobronchial HC. Past medical history included sinonasal polyposis and diffuse bronchiectasis diagnosed at the age of 12 years with a negative etiological assessment and right lower lobectomy 4 years ago. On admission, the patient had purulent sputum with no other associated symptoms, including fever, dyspnea, or hemoptysis. Chest auscultation revealed wheezing, and the patient’s oxygen saturation level was 93% (room air). The patient was treated for a bronchial superinfection. Results of microbiological investigations were negative. Flexible bronchoscopy performed after the patient improved revealed a yellowish polylobed budding formation that completely obstructed the middle lobe bronchus ( Figure 7). Chest CT-scan revealed an endobronchial lesion of fatty density in the middle lobe bronchus measuring 13 mm, responsible for minimal bronchiectasis of the middle lobe downstream, with almost total atelectasis of the latter, minimal bronchiectasis of the superior lingular segment, and sequelae of right lower lobectomy ( Figure 8). After a multidisciplinary discussion and consideration of the patient’s history, endoscopic treatment was decided. Rigid bronchoscopy revealed a smooth pale pink tumor that completely occluded the middle lobe bronchus and protruded into the bronchus intermedius ( Figure 9). Diode laser treatment was performed before mechanical ablation of the tumor using the tip of the bronchoscope. After removal, total recanalization of the middle lobe bronchus and its subsegments without endobronchial secretions was observed. Macroscopic examination revealed 2 fragments measuring 1.5 * 1.5 cm and 0.5 cm long axis, yellowish in color and with a smooth surface ( Figure 10). No complications such as bleeding, respiratory failure, or superinfection occurred. Histopathological examination of the samples stained with Hematoxylin and Eosin (H&E) showed the presence of nodules of hyaline cartilage admixed with fibrous adipose tissue, spindle cells, and myxoid stroma ( Figure 11). The diagnosis of endobronchial HC was confirmed. Flexible bronchoscopy performed 2 months after patient discharge revealed recurrence of a small yellowish formation, which reduced the lumen by 50% and was completely resected with biopsy forceps. Histological examination revealed an ulcerated bronchial mucosa seat of a hyperplastic fleshy bud, without signs of specificity or malignancy. No recurrence was observed after a follow-up period of 12 months.

## 3. Discussion

Tracheobronchial endoluminal localization of HC is rare. ^1, 2^ In a previous paper reviewing 185 cases of benign tumors of the tracheobronchial tree, HC was found in 8.1% of the cases. ^3^ HC is most frequently diagnosed in individuals between the sixth and seventh decades of life, with a higher prevalence in males. ^4^ Rare pediatric cases have been described, including one case in a three-and-a-half-month-old child. ^5^ Histologically, it is a tumor derived from peribronchial mesenchymal tissue, consisting of an absolute disorder and variable proportions of cartilage, junctional tissue, fat, smooth muscle, and respiratory epithelium. Abnormalities in the karyotype of mesenchymal cells have been reported. The most frequent rearrangement affects the 12q15 band of the HMGIC gene. ^6^ Clinical presentation due to the tracheobronchial localization of the HC is usually very noisy, either directly related to the trachea or the bronchial obstruction; in this case, the patient can be wrongly treated as asthma or COPD, as in our first patient, either in relation to its consequences, in particular obstructive pneumonia or destruction of the lung parenchyma with bronchiectasis, as in our second patient. The patient can also consult for hemoptysis if the tumor erodes a vessel. In the series by Zehani-Kassar et al., all patients were symptomatic, with general signs in four of the seven patients. ^4^ In view of the small size of the tumor at the time of diagnosis, conventional radiography is not of much help, but can show nonspecific signs such as atelectasis, pneumonia, and bronchiectasis. CT-scan is the imaging modality of choice that identifies pathognomonic signs of intralesional fat and calcifications with variable proportions. ^7^ On bronchoscopy, the appearance is highly suggestive of a benign tumor. HC presents as a well-circumscribed, polypoid, or pedunculated exophytic tumor with smooth mucosa without evidence of submucosal infiltration, usually located at the origin of a large-calibre bronchi. Its consistency is cartilaginous, but more lipomatous in the endobronchial form. ^8^ The traditional management of HC involved surgical resection. However, with advancements in endoscopic techniques, minimally invasive approaches have emerged as promising alternatives. These techniques offer several advantages including reduced morbidity, shorter hospital stay, and faster recovery times. ^3^ Surgery is currently indicated only in cases where the HC is inaccessible or when lung resection is necessary because of irreversible parenchymal damage due to longstanding airway obstruction. ^7^ In our second patient, endoscopic treatment was preferred despite the presence of bronchiectasis to preserve respiratory function in view of the history of right lower lobectomy, especially that bronchiectasis was minimal. Generally, the endoscopic approach involves rigid bronchoscopy with laser photocoagulation, electrocautery, or argon plasma coagulation, and mechanical resection. ^7^ Nd-YAG laser is the most widely used technique because of its sufficient power to vaporize tissues and its excellent coagulation effect. ^3^ However, it’s large and expensive. Diode laser, which is more compact and easier to handle, showed a clinical effect like that of a conventional Nd-YAG laser and can be a useful and safe alternative. ^9^ For residue removal, cryotherapy is a perfect choice with a lower risk of complications than photocoagulation laser. ^8^ Finally, for inaccessible segmental HC, gas jet ionized argon plasma coagulation is more suitable, allowing a noncontact treatment mode. ^8^ Generally, complications are minimal and endoscopic treatment is successful. ^3^ The prognosis of tracheobronchial HC is considered favorable. The local recurrence rate is low. In a series of seven patients published by Zehani-Kassar et al., no recurrence was noted, with a mean follow-up of 7 years. ^4^ In the series published by Casío et al., recurrence occurred in four out of 43 patients. ^10^ There is currently no consensus regarding the follow-up. The long-term follow-up did not reveal any evidence of malignant transformation. ^11^ In the 2 present cases, there was no evidence of recurrence by flexible bronchoscopy with a stable tumor residue in the first patient.

## 4. Conclusion

Owing to improvements in interventional endoscopy techniques, the endoscopic treatment of tracheobronchial endoluminal HC has become the reference treatment. Even in cases of recurrence or incomplete resection, endoscopic treatment offers favorable results. Surgery is reserved for specific indications.

### Ethics

Ethical approval was not required.

## Consent

Written informed consent was obtained from the two patients for the publication of this case report and associated images.

## Data Availability

No data are associated with this article.

## References

[1] Grabenwöger F Bardach G Mohl W : Roentgen morphology and clinical picture of pulmonary hamartochondroma. *Rontgenblatter.* 1985 Mar;38(3):72–76. 3992136

[2] Gjevre JA Myers JL Prakash UBS : Pulmonary Hamartomas. *Mayo Clin. Proc.* 1996 Jan 1;71(1):14–20. 10.4065/71.1.14 8538225

[3] Shah H Garbe L Nussbaum E : Benign Tumors of the Tracheobronchial Tree. *Chest.* 1995 Jun;107(6):1744–1751. 10.1378/chest.107.6.1744 7781378

[4] Zehani-Kassar A Ayadi-Kaddour A Marghli A : Clinical characteristics of resected bronchial hamartoma. Study of seven cases. *Rev. Mal. Respir.* 2011 May;28(5):647–653. 10.1016/j.rmr.2010.12.006 21645835

[5] Jain V Goel P Kumar D : Endobronchial chondroid hamartoma in an infant. *J. Pediatr. Surg.* 2009 Sep;44(9):e21–e23. 10.1016/j.jpedsurg.2009.06.011 19735804

[6] Fletcher JA Longtine J Wallace K : Cytogenetic and histologic findings in 17 pulmonary chondroid hamartomas: evidence for a pathogenetic relationship with lipomas and leiomyomas. *Genes Chromosomes Cancer.* 1995 Mar;12(3):220–223. 10.1002/gcc.2870120310 7536462

[7] Suzuki M Watanabe H Hashimoto M : Endobronchial hamartoma resected via bronchoscopy using high-frequency electrosurgical snare-Preoperative strategies using virtual bronchoscopy. *Radiol. Case Rep.* 2022 Nov;17(11):4232–4238. 10.1016/j.radcr.2022.08.018 36120524 PMC9471341

[8] Bouazra H Loukil M Bouzaidi K : Endobronchial hamartochondroma. *Rev. Mal. Respir.* 2013 Nov;30(9):801–805. 10.1016/j.rmr.2013.04.015 24267773

[9] Tanaka K Nakajima T Inage T : Clinical experience of transbronchoscopic laser ablation for central airway stenosis using a high-power diode laser-ten years’ experience at a single institute. *Ann. Palliat. Med.* 2022 May;11(5):1644–1648. 10.21037/apm-21-2273 35016516

[10] Cosío BG Villena V Echave-Sustaeta J : Endobronchial hamartoma. *Chest.* 2002 Jul;122(1):202–205. 10.1378/chest.122.1.202 12114359

[11] Sinner WN : Fine-needle biopsy of hamartomas of the lung. *AJR Am. J. Roentgenol.* 1982 Jan;138(1):65–69. 10.2214/ajr.138.1.65 6976714

