# Supplementary material for: Tracheobronchial endoluminal hamartochondroma resected via rigid bronchoscopy: 2 case reports of a rare entity
Source: F1000Res. 2025 Oct 14;14:76. Originally published 2025 Jan 14. [Version 2] doi: 10.12688/f1000research.159445.2 (PMC12616108; doi:10.12688/f1000research.159445.2)
Supplement: Peer review history

# Reviewer report: Reviewer response for version 2

**DOI:** 10.5256/f1000research.189414.r430247

**Published:** 2025-11-14

**Authors:** Ummi Nadira Daut

**Affiliations:** 1 Universiti Putra Malaysia, Serdang, Malaysia

For the case report, please ensure the bronchoscopy images consistently arranged orientation and suggest to put arrow to the abnormal finding (Rt vs Lt)

 Your discussion currently explains *what* happened; however lacking on explanation *why it matters* and *what is the implications for future diagnosis or therapy.*

 Detail the *on the management rationale*, explaining why you chose endoscopic management over surgery, and ****was snare electrocautery being used?

Are enough details provided of any physical examination and diagnostic tests, treatment given and outcomes?

Yes

Is the background of the cases’ history and progression described in sufficient detail?

Yes

Is sufficient discussion included of the importance of the findings and their relevance to future understanding of disease processes, diagnosis or treatment?

Partly

Is the conclusion balanced and justified on the basis of the findings?

Partly

Reviewer Expertise:

asthma phenotyping and biomarkers (FeNO, eosinophils, Vitamin D, and tocotrienols), COPD comorbidities and quality of life, tuberculosis diagnostics and public health engagement, pleural and airway intervention studies, including advanced bronchoscopy and pleuroscopy

I confirm that I have read this submission and believe that I have an appropriate level of expertise to confirm that it is of an acceptable scientific standard, however I have significant reservations, as outlined above.

# Reviewer report: Reviewer response for version 2

**DOI:** 10.5256/f1000research.189414.r430252

**Published:** 2025-11-13

**Authors:** Akesh Thomas

**Affiliations:** 1 Prisma Health Greenville Memorial Medical Campus Department of Psychiatry & Behavioral Medicine, Greenville, South Carolina, USA

The authors are describing two cases of pulmonary hamartochondroma in this case series. The cases are well defined along with their presentaiton and the treatment they have done.

 Case 1

 "The patient was subsequently hospitalized for bilateral hypoxemic pneumonitis and acute respiratory failure, which was treated as COPD exacerbation with a good evolution under usual treatment." - This sentence can be changed for better clarity. Pneumonitis is a rather non-specific description here.

 Are flow-volume loops available for this patient?

 Do not agree with the description of hypo-dense lesion on CT it would be better to specify the exact Hounsfield units if available.

 Case 2

 The flow volume loop will be interesting in this case, also if available

 Discussion

 "Clinical presentation due to the tracheobronchial localization of the HC is usually very noisy." ; Please make clarity in this sentence. Is the author saying the clinical presentation is of noisy breathing, like a stridor, or are they saying the presentation is noisy as non non-specific presentation?

Are enough details provided of any physical examination and diagnostic tests, treatment given and outcomes?

Yes

Is the background of the cases’ history and progression described in sufficient detail?

Yes

Is sufficient discussion included of the importance of the findings and their relevance to future understanding of disease processes, diagnosis or treatment?

Yes

Is the conclusion balanced and justified on the basis of the findings?

Yes

Reviewer Expertise:

Bronchoscopy, General Pulmonology

I confirm that I have read this submission and believe that I have an appropriate level of expertise to confirm that it is of an acceptable scientific standard, however I have significant reservations, as outlined above.

# Reviewer report: Reviewer response for version 2

**DOI:** 10.5256/f1000research.189414.r424923

**Published:** 2025-10-29

**Authors:** Paolo Scanagatta

**Affiliations:** 1 Hospital Eugenio Morelli, Morelli, Italy

**Summary & rationale:**

 The manuscript presents two well-documented cases of tracheobronchial endoluminal hamartochondroma treated with laser-assisted rigid bronchoscopy. The revision addresses the earlier reservations: the Introduction now includes appropriate citations; laser parameters and devices are specified for both cases; whether additional tools were used is clarified; and figure captions/panel labels have been corrected. The cases are clinically meaningful, the endoscopic approach is described clearly, and outcomes and follow-up are adequate. Overall, the article meets the journal’s standards for indexing.

**Minor, non-blocking suggestions**

1. Ensure uniform terminology throughout (use one term consistently: “hamartochondroma” vs “hamartoma”).
2. Consider adding the follow-up duration for both cases in the Abstract for immediacy.
3. Do a final proof for a few small typos/spacing issues in figure captions.

Are enough details provided of any physical examination and diagnostic tests, treatment given and outcomes?

Yes

Is the background of the cases’ history and progression described in sufficient detail?

Yes

Is sufficient discussion included of the importance of the findings and their relevance to future understanding of disease processes, diagnosis or treatment?

Yes

Is the conclusion balanced and justified on the basis of the findings?

Yes

Reviewer Expertise:

Interventional pulmonology / thoracic surgery.

I confirm that I have read this submission and believe that I have an appropriate level of expertise to confirm that it is of an acceptable scientific standard.

# Reviewer report: Reviewer response for version 1

**DOI:** 10.5256/f1000research.175169.r383632

**Published:** 2025-05-26

**Authors:** Selene Cuenca Peris

**Affiliations:** 1 General University Hospital of Castellon, Castellon, Spain

This article describes two cases of Tracheobronchial endoluminal hamartochondroma treated with rigid bronchoscopy. In addition, a review of the pathology and its approach is made and the advantages of bronchoscopy versus surgery are described. 

 This is a very interesting paper that complies with the journal's standards but requires some changes before indexing.

 In the introduction, the bibliography of the statements described is missing.

 In the description of the cases, a more detailed description of the laser technique applied (including power, time, etc.) and whether other instruments were used to remove the lesion (forceps, cryobiopsy, etc.) is missing.

 In case 2, I would change the phrase “Past medical history” to just medical history.

 In the text of Figure 2 there is no description of what projection each letter corresponds to.

 In Figure 8, the description of each letter is missing.

 Discussion, conclusions and bibliography is okay.

Are enough details provided of any physical examination and diagnostic tests, treatment given and outcomes?

Partly

Is the background of the cases’ history and progression described in sufficient detail?

Yes

Is sufficient discussion included of the importance of the findings and their relevance to future understanding of disease processes, diagnosis or treatment?

Yes

Is the conclusion balanced and justified on the basis of the findings?

Yes

Reviewer Expertise:

interventional pulmonology

I confirm that I have read this submission and believe that I have an appropriate level of expertise to confirm that it is of an acceptable scientific standard, however I have significant reservations, as outlined above.
